# Evaluation of the fullerene compound DF-1 as a radiation protector

**DOI:** 10.1186/1748-717X-5-34

**Published:** 2010-05-11

**Authors:** Aaron P Brown, Eun Joo Chung, Mary Ellen Urick, William P Shield, Anastasia L Sowers, Angela Thetford, Uma T Shankavaram, James B Mitchell, Deborah E Citrin

**Affiliations:** 1 Office of the Director, National Institutes of Health, Bethesda, MD 20892, USA; 2 Radiation Oncology Branch, National Cancer Institute, Building 10 CRC/B2-3500, Bethesda, MD 20892, USA; 3 Radiation Biology Branch, National Cancer Institute, Building 10, B2.5, Bethesda, MD 20892, USA

## Abstract

**Background:**

Fullerene compounds are known to possess antioxidant properties, a common property of chemical radioprotectors. DF-1 is a dendrofullerene nanoparticle with antioxidant properties previously found to be radioprotective in a zebrafish model. The purpose of this study was to evaluate the radioprotective effects of DF-1 in a murine model of lethal total body irradiation and to assess for selective radioprotection of normal cells versus tumor cells.

**Methods:**

*In vitro *radioresponse was evaluated with clonogenic assays with human tumor cells and fibroblast lines in the presence of varying concentrations of DF-1 or vehicle. DNA double strand break induction and repair was evaluated with immunocytochemistry for γH2AX. Lethal total body irradiation was delivered with 137Cs after intraperitoneal delivery of DF-1 or vehicle control. Bone marrow hypoxia was evaluated with piminidazole uptake assessed by flow cytometry.

**Results:**

DF-1 provided modest radioprotection of human cancer cell lines and fibroblast cell lines when delivered prior to irradiation (dose modifying factor or 1.1). There was no evidence of selective protection of fibroblasts versus tumor cells. Cells treated with DF-1 at radioprotective doses were found to have fewer γH2AX foci at 1 and 6 hours after irradiation compared to vehicle treated controls. The LD50/30 for C57Bl6/Ncr mice treated with a single 300 mg/kg dose of DF-1 pre-irradiation was 10.09 Gy (95% CI 9.58-10.26) versus 8.29 Gy (95% CI, 8.21-8.32) for control mice. No protective effects were seen with a single 200 mg/kg dose. No increase in pimonidazole uptake was appreciated in bone marrow of mice treated with DF-1 compared to vehicle controls.

**Conclusions:**

DF-1 has modest activity as a radiation protector *in vivo*. There was no evidence of selective protection from irradiation of normal versus tumor cells with DF-1.

## Background

Damage to normal tissues is a consequence of both therapeutic and accidental exposures to ionizing radiation. Total body radiation exposures can result in lethality due to hematopoetic damage, intestinal damage, and central nervous system damage. Several compounds have been described that protect tissues from exposure to ionizing radiation. The majority of agents protect against acute radiation damage are antioxidants which effectively scavenge free radicals, thus preventing indirect DNA damage, the predominant cause of cell death after exposure to ionizing radiation. The search for compounds that can reduce the deleterious effects of radiation are of interest in the setting of therapeutic radiation for cancers and in the setting of accidental or terrorism related exposures.

To categorize agents that alter normal tissue radiation response, the terms radioprotectors, radiation mitigators, and treatment have recently been adopted[1,2]. Chemical radioprotectors exert their protective effects through scavenging of free radicals[3]. A variety of compounds that act as chemical radioprotectors have been described including agents such as amifostine and other thiols,[4,5] nitroxides, [6-8] polyphenols,[9] tocols,[10] ethyl pyruvate,[11] superoxide dismutase mimetics,[12,13], melatonin and its homologues,[14] and other free radical scavengers. (reviewed in [15]). In addition to antioxidants, other compounds have been found to have radioprotective capabilities such as agents that inhibit p53 and p73 function,[16]. Checkpoint kinase inhibitors,[17] inhibitors of c-Abl,[18] and modulators of apoptosis[19] have been found to have radioprotective capabilities. (reviewed in [15]).

Carboxyfullerenes are potent antioxidants due to their free radical scavenging ability[20]. The antioxidant nature of fullerene derivatives have been exploited for a variety of disease conditions characterized by chronic inflammation or free radical generation [21-25]. Prior studies have shown that polyhydroxylated fullerenes can function as radiation protectors [26-28]. Additional modifications in the fullerene molecule side chains to enhance solubility and resultant antioxidant capacity has been undertaken[21]. One such compound is DF-1, a C_60 _dendrofullerene nanoparticle with potent antioxidant propertamifostineies[29]. DF-1 has previously been shown to improve the survival of zebrafish after exposure to ionizing radiation[28]. Little is known about the effects of DF-1 as a radiation protector in mammals such as mice. In addition, little is known about selectivity of DF-1 radioprotection in normal versus tumor tissue.

We found that human tumor cells and immortalized fibroblasts are only protected at the highest achievable concentrations of DF-1, although the magnitude of this protection was small with dose modifying factors at a surviving fraction of 0.1 of 1.1. Protection was only seen when DF-1 was delivered prior to irradiation, a finding suggestive of chemical radioprotection and consistent with the known antioxidant property. Treatment of cells with DF-1 prior to irradiation also led to a small but significant reduction in DNA double strand breaks measured by γH2AX foci at one hour after irradiation, supporting that DF-1 reduced the number of DNA double strand breaks that occurred after irradiation. We also determined that immediate pre-irradiation treatment with DF-1 can protect mice from lethal total body irradiation in a dose dependent fashion. The extent of this protection was significant at the highest dose of DF-1 delivered compared to controls, but was modest compared to previously described radiation protectors. Based on these results, our further evaluation of the radioprotective capacity of fullerenes will focus on compounds with enhanced solubility and antioxidant capacity that may provide a clinically translatable method of radioprotection.

## Methods

### Cell Lines and Treatment

The MiaPaCa2 (pancreatic adenocarcinoma) and DU145 (prostatic adenocarcinoma) cell lines were obtained from the Division of Cancer Treatment and Diagnosis Tumor Repository, NCI-Frederick (Frederick, Maryland). MRC5 (human fibroblast) were obtained from American Type Culture Collection (Manassas, VA). Cells were cultured in RPMI 1640 medium (Quality Biological, Gaithersburg, Maryland) containing 2 mM L-glutamine, supplemented with 5% (MiaPaCa-2) or 10% (DU145) fetal bovine serum (Hyclone, Logan, Utah). Cells were maintained at 37°C, 5% CO_2_. DF-1, provided by Suma Partners, was reconstituted in a 1:1 solution of DMSO and PBS and stored at -20°C. Cultures were irradiated using a Pantak (Solon, OH) X-ray source at a dose rate of 1.55 Gy/min.

### Clonogenic Assay

Cell cultures were trypsinized to generate a single cell suspension and a specified number of cells were seeded into each well of six-well tissue culture plates. After allowing 6 hours for attachment, the cells were incubated with DF-1 at the indicated concentration of DMSO (vehicle control) prior to irradiation. In some studies, DF-1 was delivered following irradiation in an alternative schedule. Following irradiation, cells were incubated for 12 to 14 days. At that time colonies were stained with crystal violet, the number of colonies containing at least 50 cells was determined, and the surviving fractions were calculated. Survival curves were generated after normalizing for cytotoxicity generated by DF-1 alone for each independent experiment. Data presented are the mean ± SEM from at least three independent experiments. Dose modifying factor (DMF) was determined from radiation survival curves by taking the ratio of radiation doses at the 10% survival level (DF-1 treated radiation dose divided by the control radiation). DMF values > 1 indicate protection.

### Immunocytochemistry

Cells grown in tissue culture chamber slides were fixed with 1% paraformaldehyde, permeabilized with 0.4% Triton X-100, and blocked with 2% bovine serum albumin (BSA) in PBS. The cells were stained with anti-γH2AX antibody (Millipore Corp., Billerica, MA), washed, and incubated with fluorescence conjugated secondary antibodies (Molecular Probes/Invitrogen,) and DAPI (Sigma-Aldrich, St. Louis, MO). Slides were examined on a Leica DMRXA fluorescent microscope (Wetzlar, Germany). Images were captured by a Photometrics Sensys CCD camera (Roper Scientific, Tucson, AZ) and imported into IP Labs image analysis software package (Scanalytics, Inc., Fairfax, VA). For each treatment condition, the total number of γH2AX foci per cell was determined in at least 50 cells.

### Mice

Ten to 12-week-old female C57/Bl6 Ncr mice (Fredrick Labs, Frederick, MD) were used in these studies. Mice were obtained at 6-8 weeks of age and caged in groups of five or less. All animals were fed a diet of animal chow and water *ad libitum*. All animal studies were conducted in accordance with the principles and procedures outlined in the NIH Guide for the Care and Use of Animals was approved by the NCI Animal Care and Use Committee.

### Toxicity Studies

Mice were weighed individually. DF-1 was delivered via intraperitoneal (IP) injection at doses of 5, 15, 35,100, 200, 300 mg/kg. All IP injections were delivered in 100 μL. Survival was assessed daily for two weeks.

### Total Body Irradiation

Mice were randomized in groups of 5 to total body irradiation at graded doses following intra peritoneal (IP) injection of vehicle control (DMSO/PBS) or DF-1 at doses of 200 and 300 mg/kg. 15 minutes following IP injection mice were transferred to plexiglass containers with holes for ventilation. Two separate containers were placed in the sample tray of the irradiator and mice were irradiated with the indicated total body doses. A 137Cs Gamma Cell 40 (Nordion International, Kanata, Ontario, Canada) was used as the ionizing radiation source. The irradiator was calibrated with thermoluminescent dosimetry chips implanted in phantom mice. The radiation dose was determined according to previously described methodology [30]. The dose rate used was 76.43 cGy/min. After irradiation mice were returned to cages for observation. Survival was assessed daily for 30 days after irradiation.

### Evaluation of bone marrow hypoxia

Mice were injected IP with pimonidazole dissolved in PBS at a dose of 60 mg/kg. Ten minutes later DF-1 (300 mg/kg) or vehicle control was delivered via IP injection. Mice were euthanized via cervical dislocation three hours following pimanidazole injection and bone marrow was harvested from both femurs. Bone marrow was immediately cooled on wet ice and flushed with PBS through a 27 gauge needle. Following centrifugation at 1200 rpm cells PBS was aspirated and cells were fixed in 4% paraformaldehyde at room temperature for 15 minutes. Following fixation cells were washed with PBS and resuspended in PBS containing 0.2% Triton-X 100 and incubated at room temperature for 10 minutes. Cells were washed once in PBS followed by resuspension in PBS containing 0.1% bovine serum albumin.

Hypoxia was assessed with flow cytometric assay using the Hypoxyprobe1 Plus Kit (HPI, Inc. Burlington, MA). Briefly, cells were reacted with anti-pimonidazole monoclonal antibody, washed, and then reacted with fluorescein isothiocyanate-conjugated anti-mouse immunoglobulin (Jackson ImmunoReserch Laboratories Inc, West Grove, PA). Positive cells were detected by flow cytometric analysis using a FACScan (BD Biosciences; San Jose, CA), with at least 10,000 cells analyzed for each set of conditions tested. Tumor cells maintained at normoxic conditions and hypoxic conditions were fixed and assayed as above as negative and positive controls). For hypoxic in vitro assays, cells were incubated for 18 hours with a closed non-vented cap.

### Statistical Analysis

In vitro experiments were repeated three times and statistical analysis was done using a student's t-test. Data are presented as mean ± SD. A probability level of P < 0.05 was considered significant. Statistical analyses of lethality studies were performed using R bioconductor package (R Development Core Team (2009) available at http://www.R-project.org). Survival of mice after irradiation was assessed by generalized logistic regression analysis (GLM). LD50/30 and 95% confidence limits were determined from GLM curve fitting of the 30 day mortality data fitted to logit curves. The doses were log transformed to improve the overall fit. Differences between survival curves were assessed by 2-tailed log likelihood ratio test of the logistic model. Prognostic relevance of the treatment in comparison to control group was assessed by Kaplan-Meier survival analysis using R statistical package. To test the difference between the survival curves, log rank test was used.

## Results

### In vitro studies

**Figure 1:**
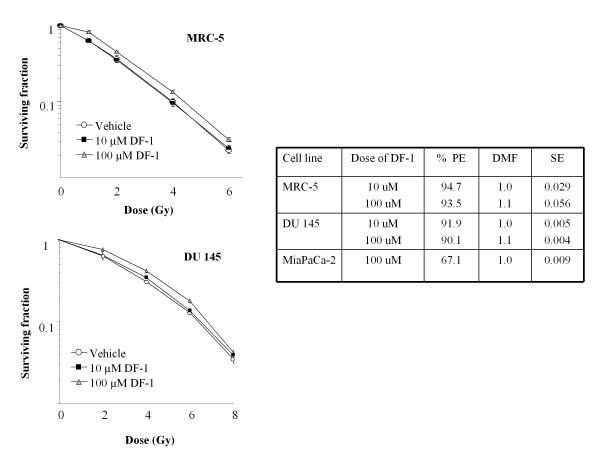
**The effects of DF-1 on cellular radiosensitivity**. Cell lines MRC5, DU145, and MiaPaCa-2 were exposed to DF-1 (100 μM and 10 μM) or vehicle control immediately prior to irradiation with graded doses of X-rays. Colony-forming efficiency was determined 10 to 14 days later and survival curves generated after normalizing for toxicity of DF-1 alone. The data represent the mean of three independent experiments. PE, plating efficiency with DF-1; DMF, dose modifying factor. Points, mean; bars, ± SE.

To determine the effects of DF-1 on tumor cell and fibroblast radiosensitivity, clonogenic survival analysis was performed in the MRC5, DU145, and MiaPaCa-2 cell lines. DF-1 was delivered at 10 μM and 100 μM final concentration immediately prior to irradiation. As shown in figure 1, DF-1 treatment at 10 μM had no effect on cellular radiosensitivity with DMFs of 1.0 for the MRC5 and DU145 cell lines. Pretreatment with 100 μM DF-1 resulted in DMF of 1.1 for both the DU145 and MRC5 cell lines. No protection was observed with MiaPaCa-2 cells at 100 μM DF-1.

**Figure 2:**
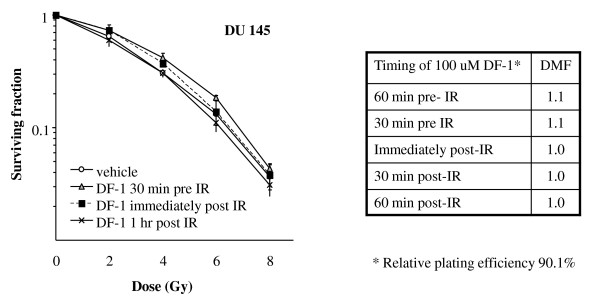
**The effects of the timing of DF-1 treatment on cellular radiosensitivity**. DU145 cells were exposed to DF-1 at 100 μM or vehicle control at the indicated times in relation to irradiation with graded doses of X-rays. Colony-forming efficiency was determined 10 to 14 days later and survival curves generated after normalizing for toxicity with DF-1 alone. The data represent the mean of three independent experiments. DMF, dose modifying factor. Points, mean; bars, ± SE.

To determine the importance of timing of DF-1 delivery on observed effect, the duration of treatment with DF-1, the duration of pre-IR treatment, and the duration of post-IR treatment were varied in single radiation dose clonogenic assays. Pre-IR treatment of up to 6 hours did not improve the efficacy of protection compared to immediate pre-IR treatment (data not shown) and post-treatment exposures of up to 16 hours did not alter clonogenic survival compared to drug removal immediately after IR (data not shown) suggesting that exposure during radiation was critical for protection. Based on these preliminary data additional complete clonogenic assays were performed to allow calculation of DMF with pre-treatment exposure times of one hour or less. Clonogenic survival analysis was performed in DU145 cells with DF-1 delivery occurring 60 minutes pre-IR, 30 minutes IR, immediately post-IR, 30 minutes post-IR, and 60 minutes post-IR. For these studies DF-1 was delivered at a final concentration of 100 μM. Relative protection with DF-1 was only observed if DF-1 was delivered prior to irradiation (figure 2).

**Figure 3:**
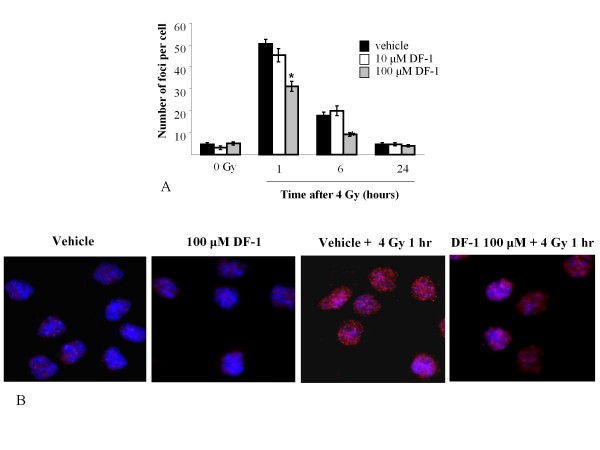
**The effects of DF-1 on DNA double strand breaks**. To investigate the effects of DF-1 on formation and repair of DNA double strand breaks after irradiation, γ-H2AX foci were evaluated by immunocytochemistry in DU145 cells. A) The number of γ-H2AX foci at 1 and 4 hrs after irradiation (4 Gy) in cells treated with 100 μM DF-1 was significantly less than that observed in cells treated with 10 μM DF-1 or vehicle alone. Columns, mean; bars, SE; *, p < 0.05. B) Representative images from stained cells.

To further investigate the cellular processes through which DF-1 protects from ionizing radiation, we focused on the DU145 cell line. DNA damage repair is an important component of radiation-induced cytotoxicity. Many radioprotectors exhibit their protective effect by scavenging free radicals and thus reducing indirect DNA damage. As a measure of radiation-induced DNA damage, we evaluated induction of nuclear foci of phosphorylated histone H2AX (γH2AX), which has been established as a sensitive indicator of DNA double strand breaks (DSBs) with the resolution of foci corresponding to DSB repair. Cells were exposed to DF-1 for 30 minutes and irradiated (4 Gy) as in the cell survival experiments, and γH2AX foci were counted at 1, 6 and 24 hrs post IR. Exposure of cells to DF-1 at 10 μM had no significant effect on the number of γH2AX foci at 1, 6, and 24 hours compared to vehicle controls (figure 3). In contrast, a significant reduction in the number of γH2AX foci per cell was observed after treatment with 100 μM DF-1 at 1 and 6 hours after IR compared to treatment with either vehicle or 10 μM DF-1, suggesting that DF-1 impacts the immediate DNA damage after irradiation. At 24 hrs the number of γH2AX foci per cell was similar in the vehicle and both DF-1 groups suggesting that DNA DSB repair was not impacted by DF-1 treatment.

### Toxicity of DF-1 via intraperitoneal injection

The maximum tolerated intraperitoneal dose of DF-1 was not reached in C57Bl6/Ncr mice. We were unable to further concentrate the agent in a suitable concentration of DMSO for in vivo studies beyond 350 mg/kg. At all dose levels, mice were observed to be hypokinetic beginning at approximately 5 minutes after injection. The duration of this effect was longer at higher doses lasting for up to 30 minutes in the 350 mg/kg group and for as short as 5 minutes in the 50 mg/kg group. This hypokinetic period was not observed in mice injected with vehicle controls. Mice treated at all doses survived through the two week observation period maintaining weight and without obvious untoward effects.

### *In vivo *radioprotection

**Figure 4:**
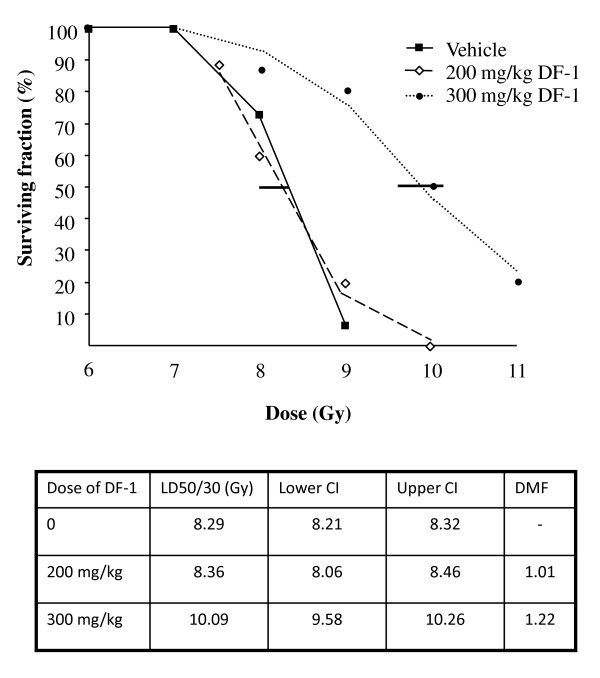
**The effects of DF-1 on 30 day survival in mice exposed to lethal irradiation**. C57Bl6/Ncr mice were randomized into three groups: DF-1 200 mg/kg, DF-1 300 mg/kg, and vehicle control. DF-1 was delivered via intraperitoneal injection in a single dose of 15 minutes prior to irradiation at the indicated doses. Mice were observed and lethality was scored at 30 days. Each group contained at least 10 mice. Horizontal bars, 95% confidence interval (CI); LD50/30, dose of radiation resulting in lethality in 50% of mice at 30 days; DMF, dose modifying factor.

**Figure 5:**
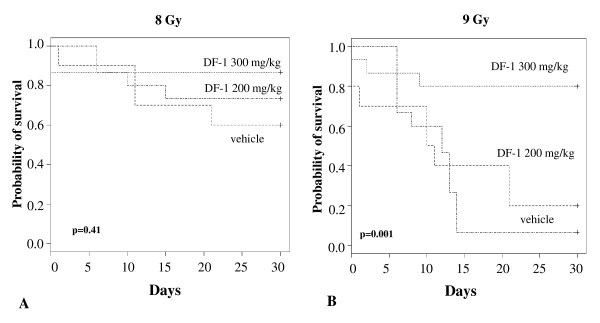
**The effects of DF-1 on survival during the first 30 days after lethal irradiation in mice**. C57Bl6/Ncr mice were randomized into three groups: DF-1 200 mg/kg, DF-1 300 mg/kg, and vehicle control. DF-1 was delivered via intraperitoneal injection in a single dose of 15 minutes prior to irradiation at the indicated doses. Mice were observed and lethality was scored daily for the first 30 days. Kaplan Meier analysis was performed for mice receiving 8 Gy (A) and 9 Gy (B) of total body irradiation. Each treatment group contained at least 10 mice.

Treatment of mice with 300 mg/kg of DF-1 by intraperitoneal injection 15 minutes prior to irradiation provided a survival advantage at 30 days. Deaths in the control group usually occurred after day 10 at doses of 8.5 Gy and lower. At doses of 9 Gy and higher deaths began as early as one week. Treatment with DF-1 at 300 mg/kg increased the 30 day survival of mice treated with total body irradiation. The LD 50/30 was determined by using doses ranging between 6 and 11 Gy with each data point representing at least 10 mice. The LD50/30 for 300 mg/kg was 10.09 Gy (95% CI 9.58-10.26) versus 8.29 Gy (95% CI, 8.21-8.32) for control mice (figure 4). This effect represents a dose modifying factor (radiation dose which caused 50% lethality at 30 days in DF-1 treated group divided by the dose of radiation which caused 50% lethality at 30 days in the control group) of 1.22. The difference in surviving fraction between the DF-1 treated mice (300 mg/kg) and the vehicle treated mice was significant (p = 0.01). Kaplan-Meier analysis revealed a significant benefit to 300 mg/kg DF-1 compared to vehicle control and 200 mg/kg at the 9 Gy dose (figure 5).

### Effects of DF-1 on bone marrow hypoxia

A number of chemical radioprotectors have been shown to induce bone marrow hypoxia, with bone marrow hypoxia correlating to protective effect[31]. We hypothesized that the hypokinetic period after DF-1 administration could possibly be related to hypotension and as a result bone marrow hypoxia. To evaluate if the hypokientic time period after DF-1 administration was associated with bone marrow hypoxia which could contribute to radioprotection, we evaluated pimonidazole uptake in marrow of mice treated with DF-1. No significant difference in the proportion of hypoxic bone marrow cells was observed with this technique suggesting marrow protection via hypoxia secondary to hypotension was not a probable secondary mechanism of action *in vivo *(table 1).

**Table 1:** The effects of DF-1 on bone marrow hypoxia measured with pimonidazole.

	Averaged mean fluorescence	Relative mean fluorescence
Normoxic cells (*in vitro*)	46.8	1.0

Hypoxic cells (*in vitro*)	89.88	1.92

Vehicle treated mice	5.18	1.0

DF-1 treated mice	5.83	1.13

## Discussion

Fullerene compounds have been studied extensively for their antioxidant properties[21,32-34]. Few studies have reported the ability of these agents to protect against exposure to ionizing radiation. As the chemical properties, such as solubility and antioxidant capacity, can vary depending on the modification of the fullerene structure,[21,35] a large number of candidate radioprotectors exist in this class that remain untested. Prior studies of fullerene compounds as radioprotectors have included an evaluation of C3, a regioisomer of water soluble carboxyfullerene, which was found to protect murine hematopoetic cells from irradiation *ex vivo*[26]. The magnitude of protection *ex vivo *was somewhat greater than that observed *in vitro *in the current study for normal cells, however these models are not directly comparable. The degree of tumor cell protection observed *in vitro *is similar with the results presented here.

The polyhydroxylated fullerene C_60_(OH)_24 _was previously evaluated as a protector of radiation and compared to amifostine in rats[27]. This study evaluated histologic measures of radiation damage but did not evaluate lethality. A recent study of the polyhydroxylated fullerene C_60_(OH)_24 _in a murine model suggested that chronic dosing of fullerene compounds can protect from lethal total body exposures[36]. This study employed dosing for two weeks prior to potentially lethal irradiation of 8 Gy. Because only a single dose of irradiation was evaluated in this study, an LD50/30 cannot be calculated, thus precluding a determination of the DMF obtained with this compound and preventing comparisons to the efficacy of DF-1.

As most lethal total body exposures are expected to occur without weeks of warning, a knowledge of the protective capacity of immediate pre-exposure dosing is important. The current study describes the ability of DF-1, a dendrofullerene compound, to protect mice from lethal total body radiation exposures. Only a modest protective effect was observed with DF-1 in the *in vitro *setting. Because of the differences in methodology of the above studies, it is impossible to adequately compare the efficacy of DF-1 to other fullerene compounds *in vitro*.

Amifostine (WR-2721) is perhaps the best studied radioprotector and has been approved for clinical use. Prior studies with amifostine have shown a concentration dependent dose modifying factor for the LD50/30 for total body exposures to ionizing radiation. The DMF for amifostine delivered as a single dose prior to a single fraction total body gamma irradiation ranges from 1.25 for 40 mg/kg to as high as 2.78 for 400 mg/kg.)[5]. This is superior to the DMF of 1.2 seen in this study with 300 mg/kg of DF-1. When considering the DMF for an agent, another important consideration is the toxicity of the agent.

The degree of toxicity of amifostine in mice correlates with the degree of radioprotection.)[5]. We observed a hypokinetic period after DF-1 administration, but these mice fully recovered, thus our maximum tolerated dose was defined by solubility limitations. It is possible that higher doses if achievable and tolerable may provide additional protection. This is also true of the *in vitro *radioprotection observed here where maximum doses were limited by solubility. Additional modifications to the fullerene compounds may enhance solubility, drug delivery, and tissue concentrations, thereby enhancing effectiveness. Given the high molecular weight of many fullerene compounds, direct comparisons of concentration may be difficult and mg dosing as opposed to μM dosing may provide a better opportunity for comparison.

We found no evidence of selectivity of normal tissue protection compared to tumor protection in our *in vitro *studies. Amifostine is known to have preferential protective capabilities in normal tissues due to a differential in the uptake in normal compared to tumor tissues [37]. It is unknown if DF-1 has this preferential uptake or other characteristics that would make it or similar compounds an attractive agent for further clinical development in the setting of therapeutic radiation.

A common mechanism of action of chemical radioprotectors is protection of DNA from indirect damage to DNA through free radical interactions. Fullerene derivatives are known to enter the nucleus of cells[38]. It is possible that they may also exert radioprotective effects through scavenging free radicals in the nucleus of cells, thereby preventing the primary lethal event of radiation, DNA double strand breaks. The protection we observed correlated with a decrease in γH2AX foci at 1 and 6 hours after radiation, suggesting that a reduction of indirect DNA damage may be the primary mechanism of action of DF-1 *in vitro*. Cai et al. reported that chronic fullerene dosing prior to total body irradiation exposure was associated with a decreased immune and mitochondrial dysfunction as well as antioxidant levels in the liver and spleen [36]. Acute exposures to fullerene compounds are unlikely to result in rapid increases in antioxidant levels in the liver and spleen selectively. However, it is likely that scavenging of free radicals and a reduction of DNA damage from irradiation is one of the mechanisms of protection in our study.

The small discrepancy between the extent of protection *in vitro *and the *in vivo *suggest that alteration of a physiologic process may be partially responsible for the observed effect. Based on the hypokinesis treated with DF-1 and the possible hypoperfusion observed in the animals treated with the combination of DF-1 and total body irradiation we evaluated the possibility that bone marrow hypoxia occurs after exposure to DF-1. Hypoxia is known to protect cells and from irradiation[39] and could be responsible for both the effect seen and the discrepancy between in vitro and in vivo effects. No difference was observed in hypoxia in the marrow of mice treated with DF-1 compared to vehicle controls suggesting that bone marrow hypoxia is not a mechanism by which DF-1 exerts is radioprotective effects.

Based on the data presented here, the fullerene compounds are of potential interest in the setting of radiation protection, although DF-1 may not be the best candidate for further development based on the limitations we described. Identification of compounds with superior solubility and anti-oxidant capacity should be undertaken in the future and evaluated in this setting. Additional explorations into mechanisms of efficacy are warranted when compounds with substantial activity are identified.

The equilibration and clearance of fullerene compounds are dependent on structure[21]. In general fullerenes are known to equilibrate rapidly after intraperitoneal delivery[21]. Clearance occurs over the course of days[21]. Concentration in liver, spleen, and bone have been reported at time points over one hour[21]. Additional modifications to the fullerene compounds may theoretically allow targeting of specific organs for protection. This may be particularly useful for organs with relatively low tolerance to irradiation such as lung, kidney, and liver.

## Conclusions

Acute pre-total body irradiation exposure to DF-1 has modest activity as a radiation protector *in vivo*. Pre-irradiation treatment with DF-1 reduces DNA double strand breaks consistent with a chemical radioprotector. There is no evidence of selective protection from irradiation of normal versus tumor cells with DF-1.

## Competing interests

The authors declare that they have no competing interests.

## Authors' contributions

DC conceived of the study, participated in the design of the study, performed the statistical analysis, and drafted the manuscript. AB assisted in drafting the manuscript, performed the in vitro work and molecular work, and assisted in the animal studies. AS and AT performed the animal work and assisted in drafting the manuscript. EC, MU, and WS participated in the design of the study and assisted in drafting the manuscript. JBM assisted in drafting the manuscript and participated in the design of the study.

All authors read and approved the final manuscript.
